# SILAC-MS Based Characterization of LPS and Resveratrol Induced Changes in Adipocyte Proteomics – Resveratrol as Ameliorating Factor on LPS Induced Changes

**DOI:** 10.1371/journal.pone.0159747

**Published:** 2016-07-20

**Authors:** Mark K. Nøhr, Toke P. Kroager, Kristian W. Sanggaard, Anders D. Knudsen, Allan Stensballe, Jan J. Enghild, Jens Ølholm, Bjørn Richelsen, Steen B. Pedersen

**Affiliations:** 1 Institute of Clinical Medicine, Aarhus University, Aarhus, Denmark; 2 Department of Endocrinology and Internal Medicine, Aarhus University Hospital, Aarhus, Denmark; 3 Laboratory for Proteome Analysis and Protein Characterization, Department of Molecular Biology and Genetics and iNANO, Aarhus University, Aarhus, Denmark; 4 Department of Health Science and Technology, Aalborg University, Aalborg, Denmark; GDC, GERMANY

## Abstract

Adipose tissue inflammation is believed to play a pivotal role in the development obesity-related morbidities such as insulin resistance. However, it is not known how this (low-grade) inflammatory state develops. It has been proposed that the leakage of lipopolysaccharides (LPS), originating from the gut microbiota, through the gut epithelium could drive initiation of inflammation. To get a better understanding of which proteins and intracellular pathways are affected by LPS in adipocytes, we performed SILAC proteomic analysis and identified proteins that were altered in expression. Furthermore, we tested the anti-inflammatory compound resveratrol. A total of 927 proteins were quantified by the SILAC method and of these 57- and 64 were significantly up- and downregulated by LPS, respectively. Bioinformatic analysis (GO analysis) revealed that the upregulated proteins were especially involved in the pathways of respiratory electron transport chain and inflammation. The downregulated proteins were especially involved in protein glycosylation. One of the latter proteins, GALNT2, has previously been described to regulate the expression of liver lipases such as ANGPTL3 and apoC-III affecting lipid metabolism. Furthermore, LPS treatment reduced the protein levels of the insulin sensitizing adipokine, adiponectin, and proteins participating in the final steps of triglyceride- and cholesterol synthesis. Generally, resveratrol opposed the effect induced by LPS and, as such, functioning as an ameliorating factor in disease state. Using an unbiased proteomic approach, we present novel insight of how the proteome is altered in adipocytes in response to LPS as seen in obesity. We suggest that LPS partly exerts its detrimental effects by altering glycosylation processes of the cell, which is starting to emerge as important posttranscriptional regulators of protein expression. Furthermore, resveratrol could be a prime candidate in ameliorating dysfunctioning adipose tissue induced by inflammatory stimulation.

## Introduction

Obesity is associated with numerous comorbidities such as insulin resistance and type 2 diabetes. Why and how obesity causes insulin resistance is currently not known. However, chronic low-grade inflammation, also called metaflammation, has been reported to be an inducer of insulin resistance and is often seen together with obesity [1]. Thus, efforts have been made to elucidate the eliciting factor of low-grade inflammation. Endotoxins or lipopolysaccharides are highly immunogenic compounds found in the cell wall of gram-negative bacteria in the gut, which has been proposed to cause metabolic endotoxemia and low-grade inflammation [2]. In favor of this, LPS plasma concentrations are elevated in humans [3, 4] and mice [2] consuming fat-enriched diets. Furthermore, LPS is associated with increased adipose tissue in humans and mice [2, 5].

LPS binds to Toll-like receptor 4 (TLR4), which is expressed by immune cells and is an important mediator of the innate immune response [6], but is also expressed in adipocytes [7]. Upon stimulation, TLR4 signals through either the myeloid differentiation factor 88 (MyD88)-dependent or MyD88-independent pathway, ultimately activating the NFκB pathway and transcription of proinflammatory cytokines and type-1 interferons. In addition, the MAPK pathway and thereby the transcription factor AP-1, which also controls the expression of proinflammatory cytokines, is activated by TLR4 stimulation [8]. Furthermore, it has been shown that adipocytes also contribute to the secretion of cytokines such as TNFα and IL6 [9, 10]. Thus, adipocytes act as active secretory cells, secreting not only adipokines, e.g. adiponectin and leptin, but also immuno-modulators, adding to development of a low-grade inflammatory state.

Resveratrol is a naturally occurring compound found in especially red grapes and red wine. Resveratrol has anti-inflammatory actions probably mainly due to its inhibitory effects on the NFκB pathway [11, 12]. Previously, resveratrol has been described to ameliorate many of the detrimental effects of high fat-feeding such as low-grade inflammation [13] and glucose intolerance [14, 15]. The precise mechanism of resveratrol is being debated, but consensus seems to revolve around increasing the activity of the intracellular deacetylase SIRT1, which has pleiotropic effects (see [16] for review) amongst these NFκB inhibition [17].

Protein expression is regulated both at the transcriptional- and translational level but can also be subject to posttranslational modifications (PTMs). PTMs are diverse and complex processes, expanding the translated proteome many fold. One of these PTMs is glycosylation of proteins. It has been estimated that more than 50% of the proteome undergo some form of glycosylation [18]. As an example, the O-Glycosyltransferases, such as the polypeptide N-acetylgalactosaminyltransferase 2 (GALNT2), recognize peptide motifs on pro-proteins and add GalNAc on serine and threonine [19]. O-glycosylation is believed to exert its PTM effect by interfering with proprotein convertases, which normally cleaves pro-proteins into mature proteins such as hormones and cytokines [20, 21]. Though very little is known about the importance of glycosylation, the few reports that have been published together with genome-wide association studies in relation to lipid metabolism [22–24], points towards huge biological impact.

By employing the SILAC method where amino acids are labeled differently by stable isotopes according to treatment regimen and perform mass spectrometry, we get an unbiased picture of the “whole” proteomic alterations. This opens for identifying and acknowledging new affected intracellular pathways important for pathophysiology which have not been previously described.

Here, we investigate the global expression alterations of LPS on adipocyte biology. It has long been known that inflammation affects several well characterized pathways such as the insulin signaling cascade [25], but how does the entire proteome respond to LPS and inflammation? And are other processes altered which could directly or indirect affect the adipocyte well-being? By using an proteomic approach, we bypass the issues of transcriptomics in relation to how much of the transcription is actually translated into biological active protein [26]. Furthermore, we investigate the effect of resveratrol, a known anti-inflammatory compound [27–29], on the LPS-mediated alteration of protein expression.

## Material and Methods

### Cell cultures and SILAC labeling

**Fig 1:**
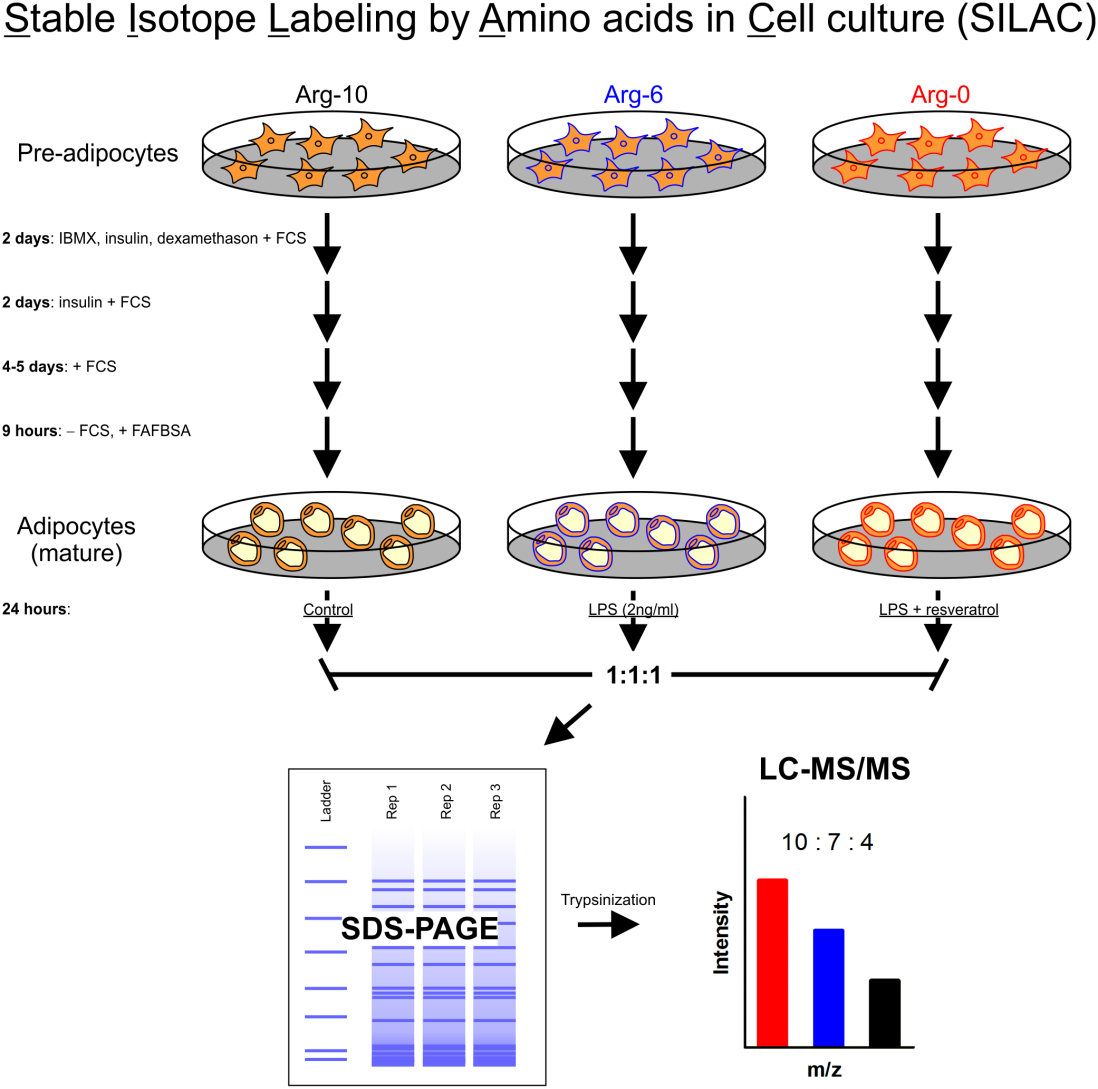
Experimental setup. 3T3-L1 cells were incubated with either heavy (Arg-10), medium (Arg-6) or light labeled arginine for 6 passages. Cells were differentiated and subjected to the treatment regimens according to labeling: Control (Arg-10 cells), LPS (Arg-6 cells) and LPS + resveratrol (Arg-0 cells). Whole cell lysates were divided by SDS-PAGE and 12 bands were cut and trypsinized before mass spectrometry analysis. Abbreviations: IBMX: 3-isobutyl-1-methylxanthine, FCS: fetal calf serum, FAFBSA: fatty acid free bovine serum albumin.

**Fig 2:**
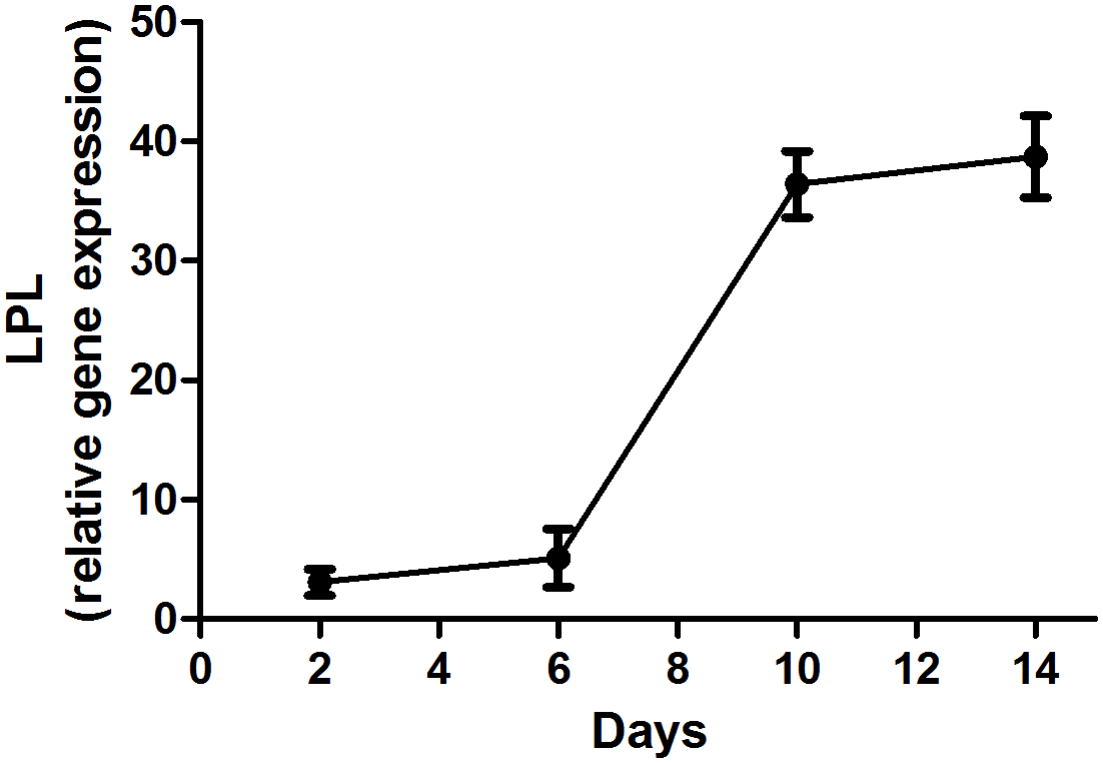
Differentiation of 3T3-L1 cells. Lipoprotein lipase (lpl) expression during the differentiation process of 3T3-L1 cells.

SILAC (stable isotope labeling by amino acids in cell culture) labeling in 3T3-L1 cells was performed as described previously [30]. Briefly, Dulbecco’s modified Eagle’s medium (DMEM; Sigma, St. Louis, MO) arginine-free medium was supplemented with heavy, medium or light labeled arginine (Fig 1). 3T3-L1 cells were grown in growth media consisting of one of the above mentioned DMEM media added 10% fetal calf serum (FCS) and 1% pen/strep for 6 passages to ensure full incorporation of labeled arginine. Growth media were changed every second day. After 6 passages, cells were grown to confluency (80%) and switched to differentiation media 2 days later (day 0) consisting of growth media added 175 nM insulin, 1 μM dexamethasone and 0.5 mM 3-isobutyl-1-methyxanthine. At day 2, cells were switched back to growth media added 175 nM insulin and 2 days later (day 4) switch to growth media. Cells were grown to day 8 after which they were serum starved for 9 hours and ready for treatment. Fig 2 shows the expression of the differentiation marker lipoprotein lipase during the days of differentiation into mature adipocytes (Fig 2). Heavy-, medium- and light-labeled arginine cells were stimulated with vehicle, 2 ng/ml LPS or 2 ng/ml LPS + 25μM resveratrol, respectively, for 24 hours. Cells were trypsinized and immediately frozen to -80°C. Cells were lysed using a RIPA type lysis buffer (10mM HEPES (pH 7.5), 90mM KCl, 1.5mM Mg(OAc)2, 1mM DTT, 0.5%v NP-40, 5%v glycerol, 0.5mM PMSF, and 10μg/mL Protease inhibitor cocktail (Sigma)). Protein concentration in the cell fraction replicates was determined using the 2-D Quant Kit (Amersham Biosciences, Piscataway, NJ). The labeled variants of each replicate were mixed 1:1:1 according to the 2-D quant results, and the proteins of the three biological replicates were separated by electrophoresis using a 5–15% SDS-Polyacrylamide gel. Each whole gel lane was excised into 12 pieces, which were individually treated using standard in-gel digestion for extracting tryptic peptides for subsequent analyses by mass spectrometry

### LC-MS/MS

Prior to Tandem MS analysis the samples were desalted essentially according to Stensballe et. al. 2003 [31] and dried by vaccuum centrifugation. The samples were redissolved in 5% formic acid before UPLC-MS/MS analysis.

The peptides were analyzed by a nanoflow UPLC (ThermoFisher Scientific; Dionex Ultimate3000/RSLC) system coupled online by a nanospray ion to an Orbitrap Q-Exactive mass spectrometer (ThermoFisher Scientific, Bremen, Germany). The peptides were loaded onto a 2 cm reversed phase Acclaim PepMap100 C18 Nano-Trap Column (ThermoFisher Scientific) with 4 uL/min in 2% buffer B (90% AcN in 0.1% formic acid and 0.005% HFBA and 98% solvent A (0.1% formic acid and 0.005% HFBA). The peptides were then separated using a 15 cm reversed phase Acclaim PepMap300 C18 column (ThermoFisher Scientific), and eluted with a linear gradient of 4% buffer B which was increased to 40% buffer B over 35 min at a constant flow rate of 300 nL/min.

The mass spectrometer was operated in a data-dependent mode to switch between full MS scans and tandem MS/MS. A top 12 mode was applied that acquired one full MS scan at a range of m/z 250–1600 at a constant resolution of 70,000 (@m/z 200), and up to 12 MS/MS scans per second at a constant resolution of 17,500 (@ m/z 200). Fragmentation was performed using higher-energy collision induced dissociation (HCD) and sequenced. Precursor ions were dynamically excluded for 30s and precursor ions from SIL pairs (+6.0204 And +10.0084) were excluded using mass tag-based exclusion.

### Animal experiments

To validate the SILAC data, we wanted to see if same changes were seen in mice treated with LPS and resveratrol. The experimental procedure has been published elsewhere [32]. Briefly, C57BL/6 mice were subcutaneously implanted with osmotic mini-pumps infusing low-dose LPS for 28 days. Furthermore, mice had free access to control diet or resveratrol diet and water throughout the treatment period. After the treatment period, tissues were harvested and immediately frozen for gene expression analysis.

### Gene expression

Gene expression analyses from tissue and cells were measured by quantitative PCR (qPCR) as previously described [32]. Primers were designed using the web-based freeware QuantPrime [33] and are listed in S3 Table. Gapdh was used as housekeeping gene.

### MS data analysis and statistics

MS raw files were processed by MaxQuant version 1.3.0.5 [34]. They were search with Andromeda [35] against a reviewed mouse proteome retrieved from Uniprot (Oct 2014, 16650 entries). Searches were done with a MS/MS tolerance of 20 ppm and 1% FDR for both peptides and proteins. Carabamidomethlyation of cysteine was set as a fixed modification, and oxidation of methionine and protein N-terminal acetylation were chosen as variable modifications. Up to two missed cleavages was allowed. One quantified peptide per protein group was allowed since further statistical analysis on the biological replicates was applied. The found SILAC ratios were log2 transformed and one-way t-test statistics was applied (H_0_:μ = 0, two-tailed). Multiple-testing correction was not applied.

### Bioinformatics

**Fig 3:**
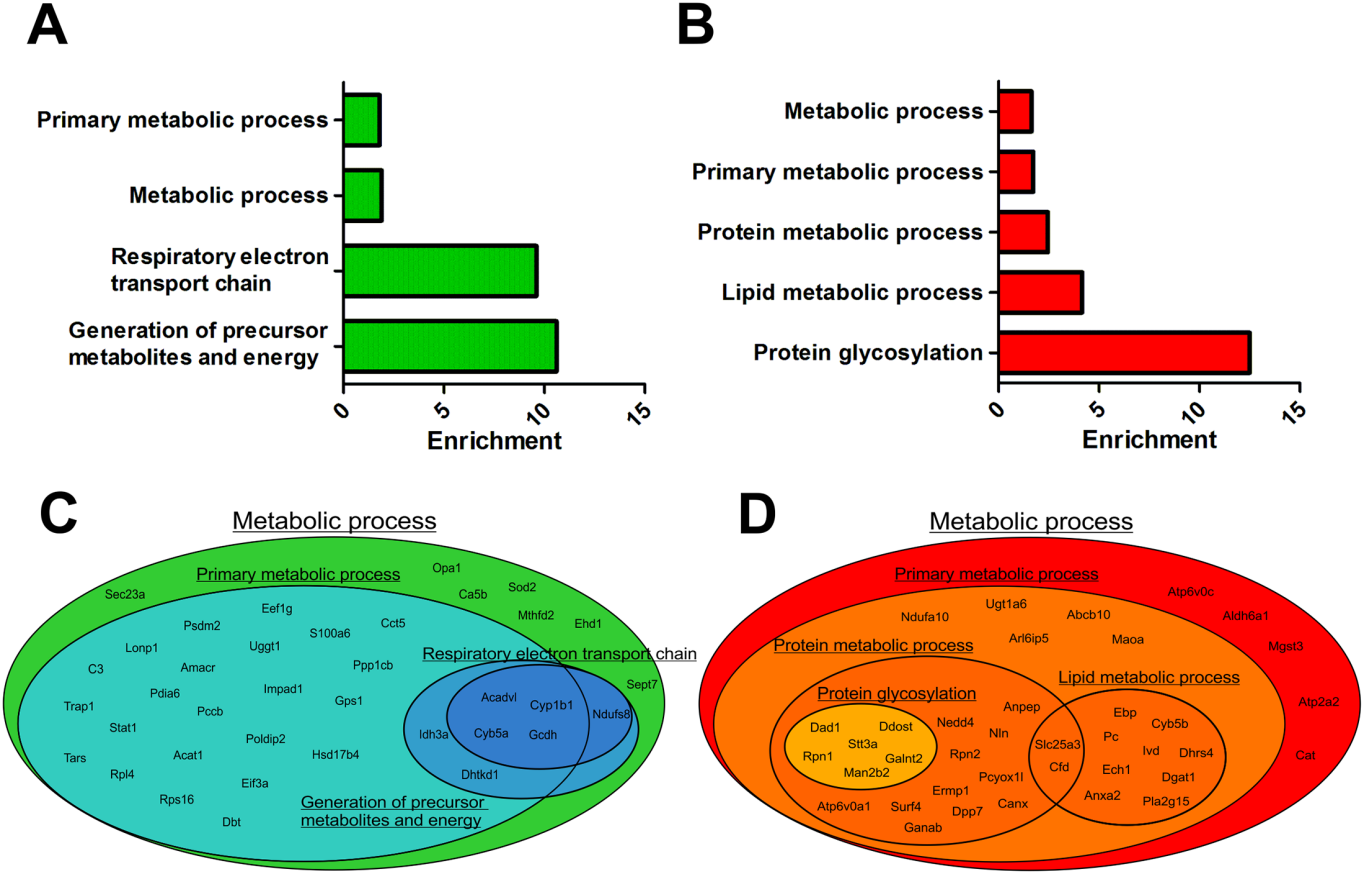
GO analysis of regulated proteins by LPS. (A) Upregulated proteins by LPS treatment belonged to the GO classes: respiratory electron transport chain and generation of precursor metabolites and energy processes. (B) Downregulated proteins especially belonged to the GO class protein glycosylation and to a smaller degree lipid metabolic processes. (C and D) Schematic overview of the distribution of upregulated (C) and downregulated (D) proteins in different GO classes represented here by gene name. Abbreviations: please see S1 Table.

GO analysis [36] of biological processes was performed on significantly negatively and positively regulated proteins. Regulated proteins were assigned to different GO classes and represented as enrichment of a given GO class against a background frequency (Fig 3A). Furthermore, protein allocation to different GO classes is shown (Fig 3B).

## Results

### GO analysis reveals LPS especially regulates metabolic processes in adipocytes

To get an overview of which biological processes were most affected by low-dose LPS in cultured adipocytes, significantly up- and downregulated proteins were analyzed by GO analysis. Overall, both up- and downregulated proteins belonged to the GO class metabolic processes. However, proteins that were upregulated, especially belonged to the metabolic GO classes of generation of precursor metabolites and energy and respiratory electron transport chain (Fig 3A and 3C). Downregulated proteins fell in the GO classes of protein and lipid metabolic processes (Fig 3B and 3D). Especially, proteins involved in protein glycosylation were highly enriched (Fig 3B and 3D). Fig 3C and 3D shows the distribution of the regulated proteins in the different GO classes.

### LPS mediated upregulation of key proteins in the interferon signaling cascade and immune-related processes

A total of 927 proteins were identified by the SILAC method. Of these, 57 proteins were significantly upregulated by LPS (S1 Table). Table 1 presents a list of the thirty most upregulated proteins. Especially proteins involved in primary anti-viral response, the interferon signal transducer STAT1 (≈ 3.5 fold) and the viral-RNA binding protein IFIT1 (≈ 7 fold), were significantly upregulated by LPS (Table 1). Furthermore, proteins in involved in host immune response such as the important complement component C3, the MHC class 1 subunit, B2M, and the antigen processing proteins H2-K1 and H2-D1/H2-Q10 (it was not possible to differentiate between the two proteins) were upregulated significantly by LPS (Table 1).

**Table 1:** Thirty most upregulated proteins.

Symbols	Full description	Fold change	*P* value
**IFIT1**	Interferon-induced protein with tetratricopeptide repeats 1	7.36	1.21E-3
**FAM129b**	Niban-like protein 1	5.27	1.50E-3
**DCTN2**	Dynactin subunit 2	4.03	1.49E-3
**EIF3A**	Eukaryotic translation initiation factor 3 subunit A	3.64	1.91E-2
**STAT1**	Signal transducer and activator of transcription 1	3.45	8.61E-3
**CYP1B1**	Cytochrome P450 1B1	3.13	2.81E-2
**COPA**	Coatomer subunit alpha	2.74	3.96E-2
**SEC23A**	Protein transport protein Sec23A	2.65	5.00E-2
**CCT5**	T-complex protein 1 subunit epsilon	2.40	1.33E-2
**C3**	Complement C3	2.39	6.42E-4
**B2M**	Beta-2-microglobulin	2.39	2.79E-2
**EHD1**	EH domain-containing protein 1	2.35	3.42E-2
**RPL4**	60S ribosomal protein L4	2.19	4.40E-2
**EEF1G**	Elongation factor 1-gamma	2.04	1.59E-2
H2-K1*	H-2 class I histocompatibility antigen, K-W28 alpha chain, K-K alpha chain, K-Q alpha chain, K-B alpha chain	1.97	2.23E-2
**OPA1**	Dynamin-like 120 kDa protein, mitochondrial	1.97	2.95E-2
H2-D1;H2-Q10*	H-2 class I histocompatibility antigen, D-B alpha chain;H-2 class I histocompatibility antigen, Q10 alpha chain, alpha chain	1.93	4.21E-2
**HNRNPAB**	Heterogeneous nuclear ribonucleoprotein A/B	1.83	1.16E-2
**PPP1CB**	Serine/threonine-protein phosphatase PP1-beta catalytic subunit	1.83	4.29E-2
**RETSAT**	All-trans-retinol 13,14-reductase	1.78	2.97E-2
**RPS16**	40S ribosomal protein S16	1.77	3.64E-2
**CYB5A**	Cytochrome b5	1.69	3.65E-2
**GPS1**	COP9 signalosome complex subunit 1	1.67	9.97E-3
**S100A6**	Protein S100-A6	1.57	4.18E-2
**RUVBL1**	RuvB-like 1	1.56	2.46E-2
**TUBB4B**	Tubulin beta-4B chain	1.56	1.04E-2
**SOD2**	Superoxide dismutase, mitochondrial	1.54	4.77E-2
**SEPT7**	Septin-7	1.53	6.41E-3
**MTHFD2**	Bifunctional methylenetetrahydrofolate dehydrogenase/cyclohydrolase, mitochondrial	1.40	9.85E-3
**DBT**	Lipoamide acyltransferase component of branched-chain alpha-keto acid dehydrogenase complex, mitochondrial	1.38	4.10E-2

*Differentiation was not possible between proteins or isoforms

### LPS downregulates adiponectin and proteins involved in glycosylation

64 proteins were significantly downregulated by LPS in adipocytes (S1 Table). Table 2 presents the thirty most downregulated proteins. The insulin sensitizing adipokine, adiponectin, was downregulated by ≈ -1.8 fold (Table 2) well in line with our previous study in human adipose tissue explants [29]. Furthermore, especially proteins involved in glycosylation of proteins such as STT3A, RPN2, DAD1, DDOST, RPN1 and GALNT2 were among the most downregulated proteins by LPS (Table 2). Finally, diglycerol acyltransferase (DGAT1), catalyzes the final step in triglyceride synthesis, and stearoyl-CoA desaturase-1 (SCD1), involved in fatty acid synthesis, together with emopamil binding protein (EBP), involved in the final steps of cholesterol synthesis, were among the most downregulated proteins by LPS (Table 2).

**Table 2:** Thirty most downregulated proteins.

Symbols	Full description	Fold change	*P* value
**GLMP**	Glycosylated lysosomal membrane protein	-2.21	9.54E-3
**SRPRB**	Signal recognition particle receptor subunit beta	-2.14	4.77E-2
**EBP**	3-beta-hydroxysteroid-Delta(8),Delta(7)-isomerase	-1.95	2.64E-2
**MYADM**	Myeloid-associated differentiation marker	-1.90	2.80E-2
**ANPEP**	Aminopeptidase N	-1.84	4.98E-2
**ITGB1**	Integrin beta-1	-1.83	4.75E-2
**NCSTN**	Nicastrin	-1.81	4.75E-2
**ADIPOQ**	Adiponectin	-1.78	2.14E-2
**ITGAV**	Integrin alpha-V	-1.77	4.49E-3
**ATP6V0C**	V-type proton ATPase 16 kDa proteolipid subunit	-1.76	2.63E-2
**DGAT1**	Diacylglycerol O-acyltransferase 1	-1.75	3.79E-3
**STT3A**	Dolichyl-diphosphooligosaccharide—protein glycosyltransferase subunit STT3A	-1.70	4.41E-2
**SLC25A3**	Phosphate carrier protein, mitochondrial	-1.69	3.88E-2
**PCYOX1L**	Prenylcysteine oxidase-like	-1.67	4.95E-2
**RPN2**	Dolichyl-diphosphooligosaccharide—protein glycosyltransferase subunit 2	-1.62	1.50E-2
**CNIH4**	Protein cornichon homolog 4	-1.61	3.44E-2
**SDHC**	Succinate dehydrogenase cytochrome b560 subunit, mitochondrial	-1.60	2.56E-2
**CFD**	Complement factor D	-1.60	2.16E-2
**DAD1**	Dolichyl-diphosphooligosaccharide—protein glycosyltransferase subunit DAD1	-1.59	2.95E-2
**CLCA2**	Calcium-activated chloride channel regulator 2	-1.59	3.68E-2
**NEDD4**	E3 ubiquitin-protein ligase NEDD4	-1.57	3.42E-2
**DDOST**	Dolichyl-diphosphooligosaccharide—protein glycosyltransferase 48 kDa subunit	-1.54	1.95E-2
**ERMP1**	Endoplasmic reticulum metallopeptidase 1	-1.53	3.85E-2
**ATP2A2**	Sarcoplasmic/endoplasmic reticulum calcium ATPase 2	-1.53	1.65E-2
**RPN1**	Dolichyl-diphosphooligosaccharide—protein glycosyltransferase subunit 1	-1.53	1.80E-2
**GALNT2**	Polypeptide N-acetylgalactosaminyltransferase 2	-1.53	4.59E-2
**TMEM43**	Transmembrane protein 43	-1.52	4.36E-2
**MAOA**	Amine oxidase [flavin-containing] A	-1.52	4.80E-2
**CAT**	Catalase	-1.51	1.55E-2
**SMARCA5**	SWI/SNF-related matrix-associated actin-dependent regulator of chromatin subfamily A member 5	-1.45	1.66E-2

### Resveratrol has a general dampening effect on LPS-affected proteins

**Fig 4:**
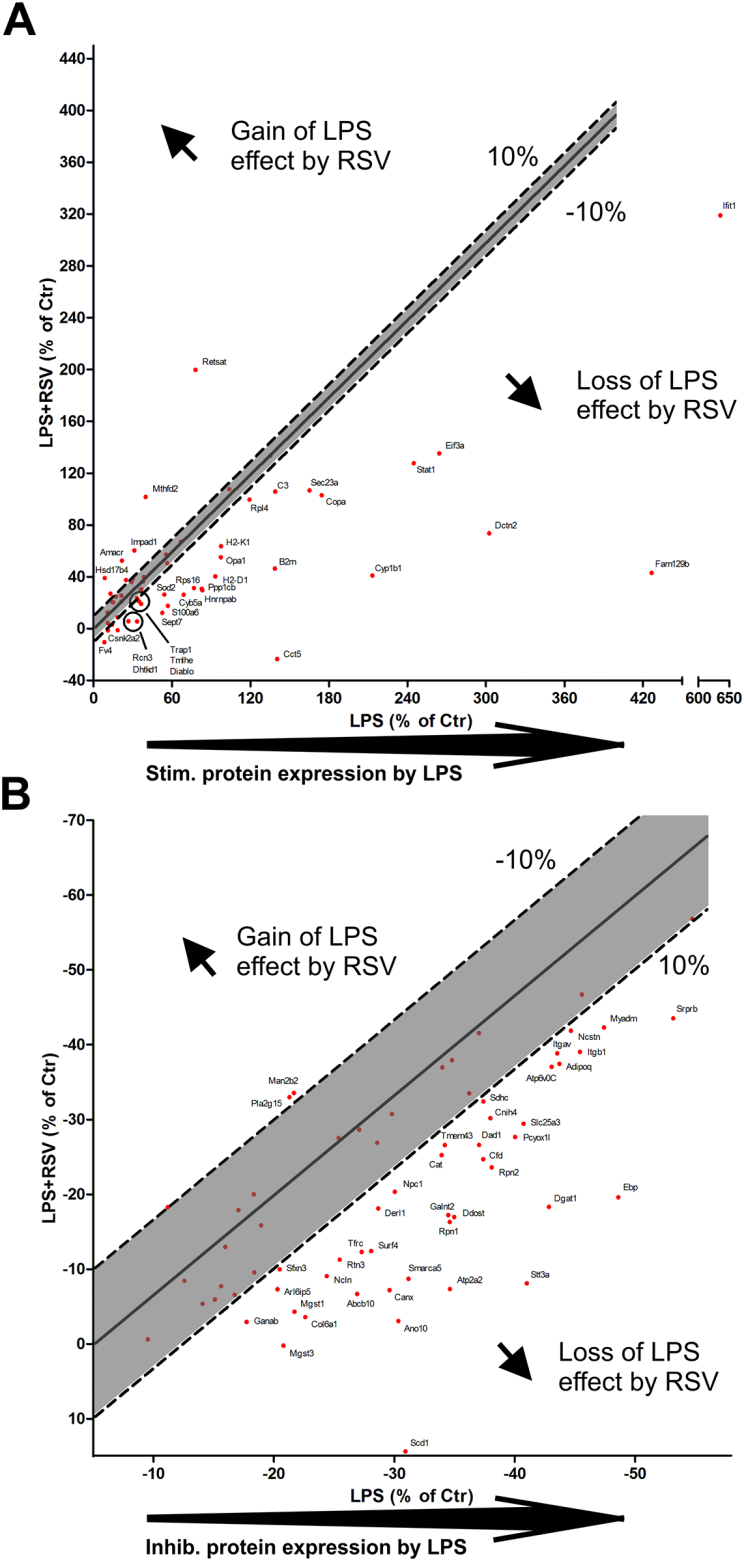
Resveratrol as ameliorating factor on LPS-induced alterations in protein expression. (A) Upregulated proteins by LPS were generally partially rescued and returned towards control expression levels. (B) Resveratrol partially reversed the downregulation of proteins induced by LPS. Abbreviations: please see S1 Table.

Fig 4 presents proteins which are regulated by more than 10% of the LPS- treatment by resveratrol. Generally, resveratrol has a rescuing effect on the proteins regulated by LPS (Fig 4A and 4B). Thus, most of the proteins that were up- and downregulated by LPS-treatment, were partially returned to control levels by resveratrol (Fig 4A and 4B). Thus, the interferon signaling proteins, IFIT1 and STAT1, were reduced from ≈ 600% and ≈ 250% to ≈ 300% and ≈ 125%, respectively, of controls by resveratrol (Fig 4A). Many of the immune response related proteins, such as C3, B2M, H2-K1 and H2-D1/H2-Q10, were reduced by resveratrol more than 10% from the LPS response (Fig 4A). Finally, some proteins, RETSAT, MTHFD2, IMPAD1, AMACR and HSD17B4, were additionally upregulated by resveratrol (Fig 4A).

Of the proteins that were downregulated by LPS, resveratrol returned the expression towards control levels (Fig 4B). Many of the glyco-proteins, STT3A, RPN2, DAD1, DDOST, RPN1 and GALNT2 were partially returned to control levels by resveratrol (Fig 4B). Also, the fatty acid and triglyceride processing proteins SCD1 and DGAT1 were reduced in severity by resveratrol. SCD1 was actually returned to a level above control level (Fig 4B). Resveratrol also showed a small ameliorating effect on the adiponectin (Adipoq) expression (Fig 4B) which fits with previous results in human adipose tissue explants [29].

### Validation of LPS and resveratrol induced effects on inflammation

**Fig 5:**
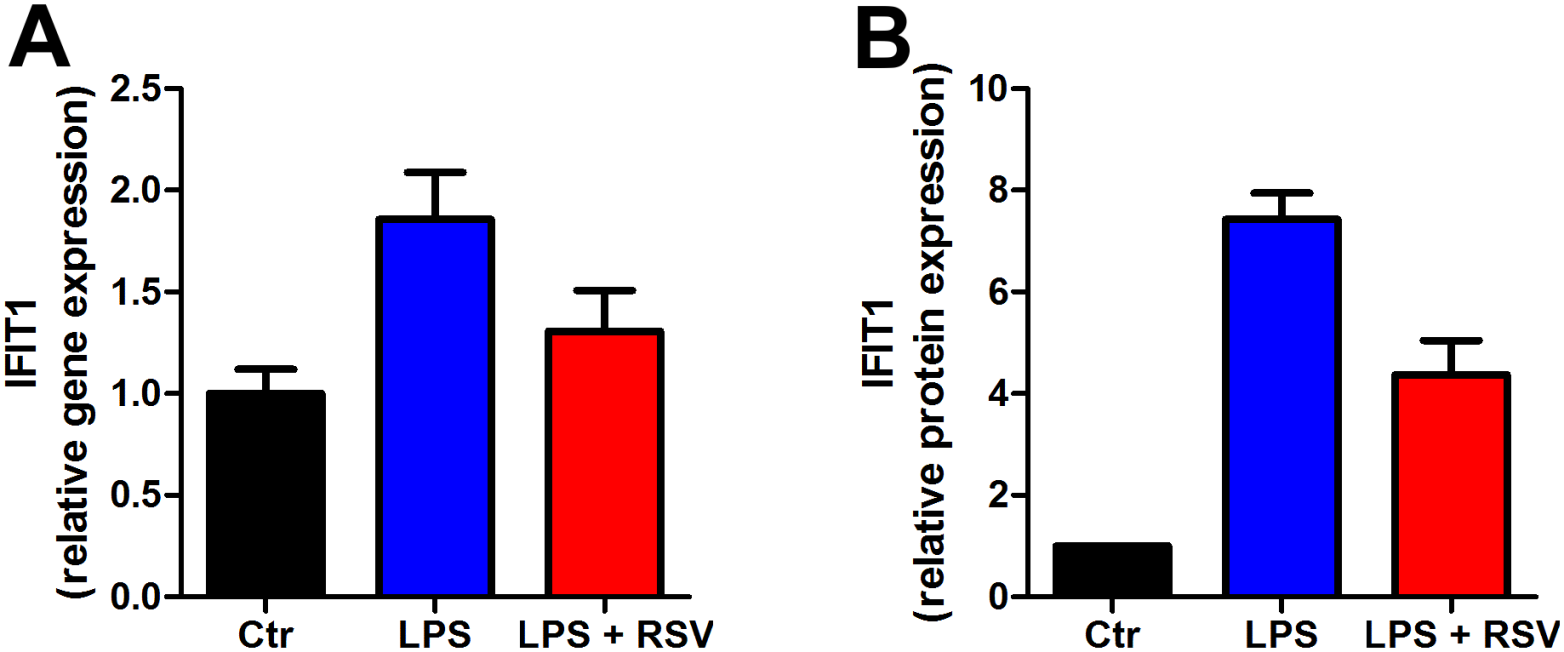
IFIT1 expression in whole adipose tissue measured by qPCR and in 3T3-L1 cells measured by and SILAC-MS. (A) Mice treated with LPS for 28 days showed increased gene expression of IFIT1, which was ameliorated by resveratrol delivered through the diet. (B) IFIT1 protein expression measured by SILAC-MS in 3T3-L1 cells incubated with LPS and resveratrol.

**Fig 6:**
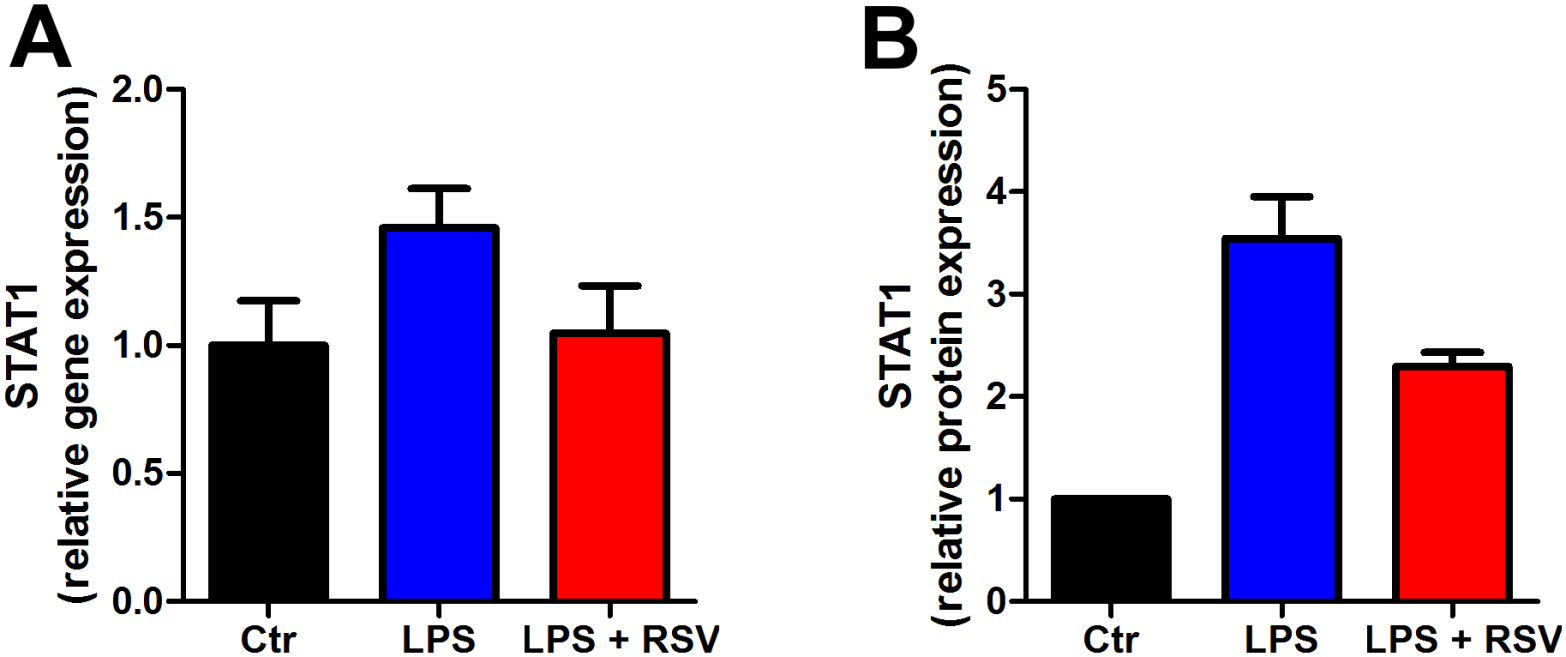
STAT1 expression measured by qPCR and SILAC-MS. (A) STAT1 gene expression measured by qPCR in 3T3-L1 cells incubated with LPS and resveratrol. (B) IFIT1 protein expression measured by SILAC-MS in 3T3-L1 cells incubated with LPS and resveratrol.

The most marked effects demonstrated with the SILAC experiment were that LPS caused robust stimulation of inflammatory proteins and an ameliorating effect of RSV. To validate these results we used qPCR to measure gene expression in whole adipose tissue as well as in vitro using 3T3 cells. To induce chronic inflammation, we treated mice with LPS in 28 days delivered through osmotic mini-pumps. Gene expression of the IFIT was elevated by LPS treatment epididymal adipose tissue but reduced close to control level by resveratrol (Fig 5A) which is similar to our SILAC data on 3T3 cells (Fig 5B). Also, STAT1 gene expression in 3T3-L1 (Fig 6A) was similar to the protein levels measured by SILAC-MS (Fig 6B). We have previously published that LPS induced an increase of the classical inflammatory biomarkers (Tnfa and Il1b) which were normalized by RSV in adipose tissue [32]. Furthermore, we measured gene expression of STAT1 in 3T3-L1 cells treated with LPS and resveratrol (Fig 6A). Again we found the same pattern in gene expression as we did for protein expression using SILAC (Fig 6B); LPS upregulated the STAT1 gene expression which was ameliorated by resveratrol (Fig 6A).

## Discussion

LPS derived from the gut microbiota has been suggested to induce low-grade inflammation as seen with obesity. In the present paper, we identify the proteomic alterations of 3T3-L1 adipocytes following incubation with low concentration of LPS. LPS increased the expression of IFIT1 and STAT1, which are involved in the interferon signaling cascade. Furthermore, proteins involved in antigen presentation and the complement system activation were upregulated by LPS. Overall, GO analysis revealed that especially proteins involved in the electron transport chain were significantly upregulated by LPS. Remarkably, LPS also caused a downregulation of proteins involved in lipid metabolism and glycosylation. Generally, resveratrol counteracted the effects of LPS, partially rescuing the protein expressions back to control levels.

It was not surprising to find that immune-related proteins were upregulated by LPS. STAT1 is an important signal transducer of interferon signaling and leads to the transcription of IFIT1. Both were among the most upregulated proteins (Table 1). Furthermore, other proteins involved antigen presentation (B2M, H2-K1, H2-D1/H2-Q10) and the complement system (C3) were upregulated by LPS treatment. STAT1 and IFIT1 upregulation has previously been seen in proteomic analysis of HeLa cells infected with reovirus [37], suggesting that despite low concentration of LPS, the 3T3-L1 adipocytes exert the same stress-response as during infection.

The GO analysis (Fig 3C) revealed that many proteins involved in the electron transport chain were upregulated by LPS. This could mean that the adipocytes have increased energy demands during inflammation, which is probably also the case. However, from Tables 1 and 2 it can be seen that various subunits of the electron transport chain complexes are both up- (NDUFA10 and SDHC) and downregulated (NDUFS8). Also, mitochondrial proteins involved in amino acid catabolism (ALDH6A1, IVD, GCDH, PCCB, DHTKD1 and DBT) and β-oxidation (ECH1, PCCB and ACADVL), show no uniform direction of expression alteration, which together with regulated expression of proteins involved in reactive oxygen species (ROS) handling (ABCB10 and SOD2), suggest that the mitochondria are under considerable amount of oxidative stress, further adding to the detrimental state of the adipocyte. From other cell types it is well-known that inflammation enhances ROS production which is important for killing pathogens, opening the inter-endothelian junction and promotion of the accumulation of inflammatory cells in the injured tissue [38].

From the GO analysis (Fig 3B and 3D) and Table 2, it was striking to see that especially proteins involved in protein glycosylation were highly and negatively affected by LPS. One of these proteins, GALNT2, has in several genome-wide association studies been linked to regulation of high-density lipoprotein cholesterol (HDL-C) and plasma triglycerides (TG) [22–24]. GALNT2 has been described to inhibit maturation of the lipase inhibitor angiopoietin-like protein 3 (ANGPTL3), which reduces the activity of the HDL-lipase, endothelial lipase [39–41]. Though endothelial lipase is mostly expressed in the liver, ANGPTL3 also inhibits lipoprotein lipase [42], which is also found in adipose tissue. More recently, GALNT2 has also been suggested to be a regulator of the soluble lipase apolipoprotein C-III [43]. Thus, most of the known function of GALNT2 is based on processing and maturation of liver proteins. Here we, however, show that GALNT2 is also expressed in white adipocytes. Obviously, the function of GALNT2 needs to be elucidated and time will show if GALNT2 is equal important in adipocyte function as seen in hepatocytes. The fact the LPS downregulates GALNT2, suggests it could play a role during the development of dysfunctional adipose tissue as seen in obesity. A recent study showed that GALNT2 glycosylation of pro-TNFα reduced the secretion of mature TNFα [44]. If this also accounts in adipocytes, we speculate it could be part of the explanation of the development of low-grade inflammation in adipose tissue. Besides GALNT2, many of the proteins/subunits in the oligosaccharyl-transferase complex, among these the catalytic subunit STT3A, responsible for N-glycosylation in the endoplasmic reticulum [45], were negatively regulated by LPS. This suggests that the general process of folding and quality control of proteins is also affected by LPS.

Surprisingly, SCD1, DGAT1 and EBP were downregulated by LPS (Table 2 and S1 Table), as LPS have previously been described to initiate obesity [2]. SCD1 is responsible for the unsaturation of stearate (C18:0) and palmitate (C16:0) to oleate (C18:1) and palmitoleate (C16:1), respectively, and as such, together with DGAT1, prepares fatty acids for incorporation into triglycerides. EBP is involved in the final steps in converting lanosterol into cholesterol. SCD1 knock-out mice have increased fatty acid oxidation and decreased triglyceride stores [46, 47]. Opposite, adipose tissue expandability and functionality during obesity have been linked to decreased “spillover” of fatty acids, resulting in decreased ectopic fat deposition and insulin resistance [48, 49]. From the latter, a correct expansion of the adipose depots is actually needed to cope with increased nutritional pressure and to avoid metabolic diseases [50]. Thus, it would seem that LPS renders a more dysfunctional adipose tissue, which could in time lead to the commencement of metabolic diseases, due to decreased expression of proteins involved in fatty acid desaturation and safe triglyceride accumulation. In favor of this, both SCD1 and EBP are under transcriptional control of transcription factors sterol regulator element-binding proteins 1 (SREBP1) and SREBP2, which are believed to be master regulators of genes involved in lipid metabolism such as fatty acid, cholesterol and triglyceride synthesis [51–54]. Thus, LPS or the secondary autocrine inflammatory effect seems to interfere with the SREBP pathway and target genes causing a detrimental state of the adipocyte.

In agreement with previous reports, we saw that resveratrol acted as an ameliorating factor on the detrimental effects of LPS [14, 15], e.g. LPS mediated downregulation of adiponectin protein expression was reversed by resveratrol (Fig 4B). This is in agreement recent data showing LPS-infused improvements of metabolic parameters by resveratrol including visceral adipose adiponectin expression [32]. Our validating of these results demonstrated good agreement between mRNA expression and SILAC data both in regards to the inflammatory effects of LPS effects as well as for the anti-inflammatory effect of RSV for selected genes both in vitro as well as in vitro (Figs 5 and 6). Generally, there is an emerging picture of resveratrol normalizing many disease states; ranging from deteriorated energy metabolism to bone metabolism [55]. Resveratrol activates the intracellular deacetylase, sirtuin-1 (SIRT1), which is also present in adipocytes [56] allowing SIRT1 mediated effects in this tissue as well. SIRT1 activation, which, among many other processes [16], has been shown to inhibit NFκB activity by deacetylating its RelA/p65 subunit and thereby hinders transcription [17]. Given that many of the effects induced by LPS are caused by increased NFκB activity, SIRT1-mediated inhibition is probably of importance.

We present here data of the global proteomic alterations in adipocytes by LPS. Adipose tissue inflammation is a key hallmark in the development of metabolic syndrome. LPS altered expression of proteins involved in metabolic processes such as glycosylation, respiratory chain transport and lipid metabolism, suggesting dysfynctioning adipocytes. Resveratrol rescued the protein alteration induced by LPS across many different metabolic processes, which suggest a general ameliorating effect. Thus, we suggest resveratrol could be a potential candidate in ameliorating some of the detrimental effects, e.g. adipose tissue inflammation, seen with metabolic syndrome.

## Supporting Information

S1 Table Total list of proteins either up- or downregulated by LPS. (XLSX)

S2 Table Total list of identified proteins by SILAC-MS method and the individual ratios of biological replicates. (XLSX)

S3 Table List of primer pairs used. (DOCX)
