# Supplementary material for: SILAC-MS Based Characterization of LPS and Resveratrol Induced Changes in Adipocyte Proteomics – Resveratrol as Ameliorating Factor on LPS Induced Changes
Source: PLoS One. 2016 Jul 20;11(7):e0159747. doi: 10.1371/journal.pone.0159747 (PMC4954707; doi:10.1371/journal.pone.0159747)
Supplement: S3 Table — (DOCX) [file pone.0159747.s003.docx]

**Table S1**

| Gene | Primer | Secuence (5’ -> 3’) |
| --- | --- | --- |
| Lpl | Forward | CTCCAGAGTTTGACCGCCTTC |
|  | Reverse | TCTCTTCCCGCGTCTGCT |
| Stat1 | Forward | TGACAAAGACCACGCCTTTGGG |
|  | Reverse | AAGCTCCATCGGTTCTGGTGCTTC |
| Ifit1 | Forward | AGCAGAGAGTCAAGGCAGGTTTC |
|  | Reverse | TGGTCACCATCAGCATTCTCTCC |
| Gapdh | Forward | TTGATGGCAACAATCTCCAC |
|  | Reverse | CGTCCCGTAGACAAAATGGT |

Primer sequences
